# Exploring disparities in self-reported knowledge about neurotechnology

**DOI:** 10.1038/s41598-025-00460-1

**Published:** 2025-05-27

**Authors:** Sebastian Sattler, Guido Mehlkop, Alexander Neuhaus, Anna Wexler, Peter B. Reiner

**Affiliations:** 1 Faculty of Sociology, Bielefeld University, Bielefeld, Germany; 2 Pragmatic Health Ethics Research Unit, Institut de Recherches Cliniques de Montréal (IRCM), Montreal, Canada; 3 Faculty of Economics, Law and Social Sciences, University of Erfurt, Erfurt, Germany; 4 Institute for Planetary Health Behaviour, University of Erfurt, Erfurt, Germany; 5 Department of Medical Ethics and Health Policy, University of Pennsylvania, Philadelphia, USA; 6 Department of Psychiatry, University of British Columbia, Vancouver, BC Canada

**Keywords:** Public health, Translational research, Spinal cord injury, Patient education, Rehabilitation, Medical ethics, Rehabilitation

## Abstract

With advances in neurotechnology and its use for medical treatment and beyond, it is important to understand the public’s awareness of such technologies and potential disparities in self-reported knowledge, because knowledge is known to influence the acceptance and use of new technologies. This study utilizes a large sample (*N* = 10,339) to depict the existence and extent of self-reported knowledge of these neurotechnologies and to examine knowledge disparities between respondents. Results show that most respondents self-reported at least some knowledge of ultrasound and electroencephalography (EEG), but limited knowledge of BCIs. Prior use, being a healthcare professional, and health literacy increased the odds of self-reporting some knowledge. Also gender and age disparities exist. These findings may help identify uninformed groups in society and enhance information campaigns.

**Supplementary Information:**

The online version contains supplementary material available at 10.1038/s41598-025-00460-1.

## Introduction

Neurotechnology embraces devices and interventions targeting the brain and the nervous system such as ultrasound, electroencephalography (EEG), functional magnetic resonance imaging (fMRI), brain and spinal cord stimulation, or brain-computer interfaces (BCIs)^1–3^. These technologies are used within and outside the medical context for various purposes including diagnosis (e.g., attention deficit hyperactivity disorder or epilepsy), treatment (e.g., Parkinson’s disease or dystonia), restoration (e.g., after spinal cord injury), or enhancement (e.g., of learning and memory in work, education, or recreation), so it is likely that most people will need to use them at some point in their lives^4–7^. Some of these technologies are also used in do-it-yourself applications^8–10^. Neurotechnologies are currently being further developed with great hopes—including financial revenues for tech-companies and opening up other fields of application^11–15^. However, neurotechnology also bears risks, like changing the brain in unpredictable ways, stigmatization of users and refusers, patient autonomy, data protection, or responsibility for device failure^14,16,17^. These implied potentials and risks have propelled a debate about various ethical, social, and legal challenges.

Public perceptions of neurotechnology are influenced by a history of distrust in novel medical treatments, which have arisen in part from unethical medical practices such as psychosurgery^18,19^. This distrust is further shaped by media portrayals and discussions in social media and film, which sometimes sensationalize or erroneously present advancements or issues, leading to misconceptions^20–24^. As several of these technologies are already in use across different spheres of life, it is crucial to better understand whether the public is aware of neurotechnology and how much they believe they know about it.

In theories related to health decision-making and technology acceptance and use, an individual’s knowledge or familiarity with an issue at hand is a relevant direct predictor or can be seen as an antecedent of core theoretical factors. For example, in the Health Belief Model^25^, knowledge is one of the modifying factors of individual health beliefs such as perceived self-efficacy and barriers; in the Social Cognitive Theory, a person’s ability to perform a behavior is a function of knowledge^26^; and in the Unified Theory of Acceptance and Use of Technology^27^, knowledge is a facet of the facilitating conditions. Concerning decision-making about neurotechnology, it has been also been speculated that lacking familiarity with brain stimulation explains the cautiousness to use such technology^28^. First research on neurotechnology, for example, by Williams et al.^29^ found that lower BCI knowledge was associated with an unwillingness to participate in BCI insertion. However, another survey found a statistically insignificant (but slightly negative) relationship between BCI knowledge and concerns about BCIs^14^. A small-scale survey of patients with obsessive–compulsive disorder found a statistically insignificant (but positive) trend between prior knowledge and being more positive to undergo deep brain stimulation (DBS) treatment^30^. It was also found that greater familiarity with brain implants for enhancement was associated with greater willingness to use such technology for oneself^31^, although it is unclear whether the familiarity drives this desire or whether favoritism towards the technology increases information seeking. Another study found that familiarity with the mechanisms of action of invasive stimulation techniques partially mediated the relation between this mechanism and willingness to use^32^.

A lack of knowledge can, however, increase the risk of biased information searching, make it more difficult to assess the credibility of new information, and lead to misconceptions—which, in turn, might result in even less factual knowledge and spreading misinformation^33–35^. Depending on the misconceptions, they may also reduce patient acceptance of the technology, as has been suggested for BCIs^36^, while they may also create unfounded enthusiasm and false hopes including uncritical use, as has been criticized based on media portrayals^20–22^. Moreover, knowledge can affect application experiences and outcomes of applications (e.g., placebo effects versus treatment effects)^37^.

Thus, limited knowledge can be critical when individuals need to decide about the use of neurotechnology, for example, when using consumer devices such as brain stimulation. In the context of such nonmedical applications, limited or biased knowledge can be particularly problematic as individuals could be more susceptible to false information or commercials with exaggerated promises. Moreover, nonmedical applications without the involvement of healthcare professionals could result in careless use. While many people gain detailed information about treatment from their specialists, they may also turn elsewhere when evaluating the presentation of diagnostic results or treatment options based on their physician’s suggestions (including in the context of informed consent). Moreover, physicians’ time constraints may not allow them to devote sufficient time and care to answer all their patients’ questions. In addition, patients may be reluctant to disclose gaps in their knowledge and understanding, which could lead to the rejection of neurotechnology or anxiety during application. Thus, prior knowledge of neurotechnology (including from different sources) can facilitate understanding of relevant information and inform decision-making. A survey of Parkinson disease patients showed that only 4% reported receiving information from their primary care physician, while 31% reported the neurologist as the primary information source^38^. This may also be due to general practitioners having less knowledge than specialists^39^ or the propensity of general practitioners to refer patients to specialists for the provision of more detailed neurological information. Thus, having more valid knowledge increases patient autonomy as it reduces information asymmetries between patients and physicians—who may also not be fully knowledgeable about neurotechnologies, may hold biases against neurotechnology^40^, may be influenced by neurophobia, which may affect their treatment decisions and referral pattern and health care practice^41^, or may have certain financial interests in suggesting certain diagnostic or therapeutic means (e.g., if trained in applying neurofeedback, they may suggest it). Moreover, knowledge may influence both patient self-referrals and reacting to patient-to-patient referrals.

In theories of stigma, such as the familiarity hypothesis, knowledge has been described as a relevant factor that can decrease, but under certain circumstances also increase, stereotyping of individuals as well as prejudice and discriminatory behavior toward them^42^. The phenomenon of neurophobia observed among future physicians and practitioners and their associated discomfort in studying neuroscience subjects has been linked to a poor understanding of these subjects and a suboptimal comprehension^38,39,43^. The general public’s knowledge about neurology and neurotechnology may be even lower than that of medically trained individuals, which may affect their interaction with individuals who require or already use neurotechnology. Initial evidence, not investigating the role of knowledge, evidences public stigma associated with the refusal of neurotechnology^44^.

However, there is limited research on the knowledge of certain neurotechnologies among different stakeholder groups, particularly the general public. One study describes a lack of neuromodulation knowledge in rural family medicine residents^45^. Upon entering medical school, students, who are the future physicians, lack knowledge about deep brain stimulation, while the knowledge gain during training was moderate^43^. In a survey, the majority of psychiatrists reported knowledge of DBS for oppressive-compulsive disorder, but less so cognitive behavioral therapy psychotherapists, while patients seem to have limited knowledge^30^. Another study found that only 6% of US adults reported having heard “a lot” about brain chip implants to improve cognitive abilities, 32% “a little”, and 61% “not at all”^31^, which hardly changed five years later^46^. A trinational survey of the general publics in Canada, Spain, and Germany also shows that the public has little knowledge concerning BCIs^14^. In another representative survey of the general public in Germany, less than one third of participants reported knowing what a BCI is^47^. Also limited knowledge was found in a survey in France^36^. Although first studies are beginning to describe the prevalence of knowledge about neurotechnology, they have hardly simultaneously examined knowledge about multiple neurotechnologies.

Only few studies have examined individual differences in neurotechnology knowledge. For example, self-reported knowledge about BCIs was particularly low among respondents who identified as “female,” had less formal education, or reported lower levels of religiosity^14^. Arhan et al.^48^ found that experience with EEG with their children increased mothers’ knowledge of EEG. Higher income levels were associated with greater knowledge about EEG. Medical professionals also reported more knowledge of DBS use than patients^30^. Given the relatively few studies, inconsistent findings, and understudied characteristics, further research is needed to identify groups of individuals who lack or have limited knowledge. It might thereby be of particular importance to examine differences across sociodemographic groups (e.g., gender and age), prior use, indicators of increased ability to know and understand such technology (e.g., working in a health context), health indicators, and different values and worldviews. This would be relevant since previous research on neurotechnologies^14,31,32,49,50^ and other technologies^51–53^ suggests that these factors might be associated with direct or indirect experience with neurotechnology, indicators of current need or desire to use, or ideological aversion to using such technology (as it may be perceived as transgressing human boundaries or “playing God”).

Thus, a description of the public´s self-reported knowledge, as well as an understanding of which groups in the population lack knowledge, is theoretically relevant, as knowledge may be an important predictor of health-related decisions, whether individuals respond with stereotyping and discrimination to neurotechnology users and refusers, as well as a mediating factor between other variables in decision-making models. It could also be of practical use for informing the professional and public debate and health education—including debunking myths or misconceptions that create technology avoidance and reduce exaggerated hopes, but also informing about beneficial uses^16,30,47,48^.

### The current study

This study utilizes a large nationwide sample of the adult population in Germany to identify the public that has never heard of six neurotechnologies, namely ultrasound, EEG, fMRI, brain stimulation, spinal cord stimulation, and BCIs (the reasons for selecting these neurotechnologies are given below). It also depicts the extent of self-reported knowledge among those with at least limited knowledge. Moreover, to better understand the disparities in knowledge, it examines associations with sociodemographic characteristics (e.g., gender and age), the presence of prior experience of using neurotechnology for treatment, diagnosis, or other use purposes, an elevated ability to understand such technology (e.g., health literacy or working in a health or care-related job), indicators of an elevated likelihood of current or future need of neurotechnology (e.g., for their mental and physical health or to cope with stress), as well as different values and worldviews that more likely lead to a rejection of neurotechnology (e.g., religiosity). This investigation occurs within the context of an aging society (more than a quarter of people in Germany are 60 and older)—a trend particularly notable in many Northern Hemispheric countries^54^. As populations age, there is a corresponding increase in age-related cognitive and physical decline as well as a need for care^55^. Moreover, German society is one in which high levels of stress are prevalent^56,57^. Thus, this context suggests a growing need for neurotechnological solutions to prevent, diagnose, or treat diseases; to restore abilities; and to enhance functioning. Germany, as a high-income country^58^, possesses a healthcare system capable of supporting the adoption of neurotechnologies, including newer and more expensive innovations. The mandatory health insurance system ensures comprehensive health coverage for all residents, facilitating access to these neurotechnologies. Furthermore, the availability of economic resources—including a high purchasing power in senior citizens^54^—also allows for the exploration of neurotechnology for non-medical purposes. However, access is just one factor influencing the adoption of neurotechnology. By investigating public awareness and the extent of self-reported knowledge about neurotechnology as well as disparities therein, this study leverages the understanding of key variables in various models of health decision-making and technology acceptance, and provides practical insights into a factor malleable through health education or other interventions.

## Methods

### Sample

We use data from the fourth wave of a nationwide web-based study called *ENHANCE* for which adults (18 and older) were recruited offline and nationally representative with respect to gender, age, education, and federal state for the adult residential population in Germany with Internet access (which is 95% of all households in Germany). We invited 28,567 individuals to wave four, including individuals who declined to participate in waves two and/or three, and 10,104 individuals for the refreshment sample (both inclusions were made to compensate for demographic imbalances due to the selective participation of hard-to-reach participants). Thereof, 13,452 individuals consented to participate (47.1%), and 12,168 completed the voluntary survey (90.1%) for which data were pseudonymized. Completers received bonus points as incentive (worth approximately $3.00) redeemable to vouchers, charity lottery tickets, or donations to UNICEF. Our analytic sample comprises 10,339 individuals who responded to all variables used in the analysis (48.8% female, mean age: 49.93 years; Table 1 for more descriptive statistics). The study was conducted in the German language and approved by the ethics committee of the University of Erfurt (reference number: EV-20220830). Our work aligns with the Code of Ethics of the American Sociological Association (ASA) and although our study is not a medical study, we adhere to the Code of Ethics of the World Medical Association (Declaration of Helsinki) to protect human research participants.

**Table 1:** Descriptive statistics (*N* = 10,339).

	Mean/proportion	SD	Minimum; maximum
Experience with neurotechnology^a^			
Diagnosis	18.3%		
Treatment	3.2%		
Other	1.9%		
Healthcare profession			
No profession/other profession	93.3%		
Yes	6.7%		
Health literacy			
Untransformed	3.28	0.66	0;4
Standardized to 0–1 interval	0.82	0.16	0;1
Physical health			
Untransformed	6.23	2.16	0;10
Standardized to 0–1 interval	0.62	0.22	0;1
Diagnosed mental illness			
No	62.8%		
Yes, still in treatment	12.7%		
Yes, no longer in treatment	24.5%		
Stress			
Untransformed	1.48	0.8	0;4
Standardized to 0–1 interval	0.37	0.20	0; 1
Religiosity			
Untransformed	2.98	3.13	0;10
Standardized to 0–1 interval	0.3	0.31	0;1
Sex			
Male	51.2%		
Female	48.8%		
Age			
18–24	4.5%		
25–34	17.8%		
35–44	17.5%		
45–54	16.1%		
55–64	24.7%		
65–74	15%		
75–95	4.5%		
Ethnicity			
Non-German	5.4%		
German	94.6%		
Education			
Secondary and below	11.4%		
Secondary II	29.7%		
University entrance qualification	21.7%		
Tertiary	37.2%		
Household equivalence income			
Low (< 60% median)	14.9%		
Medium	80.4%		
High (> 2*median)	4.7%		
Children			
None	38.1%		
One or more	61.9%		
Place of residence			
Rural	28.9%		
Urban	71.1%		

^a^Multiple responses were possible.

### Measures

*Self-reported knowledge about neurotechnology:* We aimed to assess a broad range of awareness and self-reported knowledge across the different ways in which individuals may have encountered neurotechnologies, including consumer devices, routine neurological care, and via novel devices mentioned in the media. Our choice of neurotechnology includes both specific technologies as well as broad categories of technologies. In selecting the technologies to be assessed, we had to accept trade-offs between the technologies themselves and the added constraint of what we could reasonably ask about in a single survey. We were also faced with the fact that many technologies utilized in one domain (i.e., consumer) are also used in another (i.e., medical). We did not explicitly address the issue of invasive versus non-invasive neurotechnologies, as we considered this distinction to be one that specialists in the field might make (even if there is disagreement among them cf.^59^). We selected brain-computer interfaces and spinal cord stimulation because they are two types of neurotechnologies that have received extensive media coverage in recent years, and the media commonly uses this terminology to describe them (spinal cord stimulation is a specific technique, but it has received extensive media coverage in the context of supporting paralyzed individuals to walk). We chose EEG and brain stimulation because these are the technologies used by consumer neurotechnology devices^9,60^. We acknowledge that EEG is a specific neural recording technique, and that brain stimulation encompasses a multiplicity of techniques (transcranial alternating current stimulation (tACS), transcranial current direct stimulation (tDCS), transcutaneous vagus nerve stimulation (tVNS), among others). However, we chose EEG because (a) to our knowledge, there are no non-EEG neural recording devices currently marketed to consumers; and (b) we felt that it would be more likely that individuals would be familiar with the term EEG rather than terms such as “neural recording” or “brain sensing.” Likewise, we chose the broad category of brain stimulation because consumer devices employ a range of techniques but are often inexplicit about the specific form of neurostimulation being used. We recognize that EEG and brain stimulation are also used in clinical care, and in this context the latter can encompass invasive forms of neural stimulation. The final two technologies, fMRI and ultrasound, were chosen because we sought to include additional neurotechnologies that the public may have encountered during routine medical care. Thus, respondents were asked to self-report their knowledge about six neurotechnologies, including ultrasound, EEG, fMRI, brain stimulation, spinal cord stimulation, and BCIs by using the question “How would you rate your knowledge of the following neurotechnologies?”. Respondents indicated their knowledge using a scale from “never heard of it” [value 0], “very little knowledge” [1] to “very much knowledge” [7]. Participants could also choose a non-scaled response option (“no response”). For each neurotechnology, two indicators have been created to first, examine factors influencing the complete absence of knowledge [0] versus any knowledge [1] and to second, examine factors influencing the extent of knowledge indicated by values ranging from 1 to 7, excluding respondents indicating no knowledge at all. In an experimental pre-study (see Pre-Study in the Online Supplements), we have examined whether it makes a difference to either provide the respondents with information about the areas of application, functioning, and names of devices (in case of consumer availability of the technology) vs. providing only the name of the neurotechnology. We could hardly detect any differences of such information on whether respondents remembered if they had heard about the six neurotechnologies, as well as on the extent of knowledge, while survey time was substantially lower without such information. Thus, providing such information seemed to be of limited value compared to no information, while respondents may have easily categorized themselves without such cues. Therefore, and in the interest of reducing the burden of longer survey times and survey costs, this study assessed self-reported knowledge about neurotechnology without further information on the neurotechnology.

*Experience with neurotechnology:* Respondents were asked whether and, if so, for what reason neurotechnologies were used by or on them for medical purposes (e.g., for the prevention, diagnosis, or treatment of disease) or for other purposes (e.g., restoration of declining physical/mental abilities, improvement of physical/mental abilities without medical need or to control a machine at work or in other contexts) cf.^9^. Multiple responses were possible. Based on the answers, three binary variables indicating experience regarding diagnosis, treatment, or other (combining all answers for other purposes given the low frequencies) were created [coded 1] vs. no such experience [0].

*Health literacy:* Based on the Health Literacy Screening Questions^61^ which are derived from the Short Test of Functional Health Literacy in Adults (S-TOFHLA)^62^, we assessed health literacy with three questions about the frequency of seeking help or having problems finding out about the state of health (assessed on a scale from “never” [0] to “very often” [4]) and a question about the confidence in filling out medical documents (“very sure” [0] to “not sure at all” [4]). A mean was computed and then the scale was standardized on a 0–1 interval whereby higher scores indicate high literacy.

*Physical health:* We used one question from the German version of the Flourish Index^63,64^ to measure subjective physical health (“In general, how would you rate your physical health?”) with response options ranging from “poor” [0] to “excellent” [9].

*Stress:* We used three items of the German version^65^ of the Perceived Stress Scale^66^ to measure chronic stress (exemplary item: “In the last 12 months, how often have you felt that you were unable to control important things in life?”). Response options ranged from “never” [0] to “very often” [4]. Internal consistency was satisfactory (Table 1). A mean was computed, and the scale was standardized to a 0–1 interval whereby higher values indicate more stress.

*Religiosity:* Religiosity was assessed using the question “How important is religion to you?” with the response option “not important” [0] to “very important” [10] cf.,^67^.

*Sociodemographic characteristics:* We assessed the respondents’ sex, age, education, and children (including biological children, adoptive, stepchildren). Moreover, to assess ethnicity, respondents indicated the community to which they have the most and second most strong sense of belonging to. If an answer to either of these questions was ‘German’, this was coded German [1] and otherwise not [0]. Household equivalence income was measured through the respondents’ estimated household net income using an open-ended question, and when no answer was provided, income categories were shown cf.,^68^. We used the mean value for income categories and, for the last open-ended category (“20.000 and more”), the value 20.000 was used^69^. The equivalence income was computed by considering the number of household members with the OECD-modified scale^70^. Thereby, the first adult received a weight of 1, each additional adult was weighted 0.5, and each child (under 14 years) was weighted 0.3. Finally, income was divided by the weight. Based on an open-ended question concerning the respondent´s job and using the classification of occupations which refers to the International Standard Classification of Occupations—ISCO, healthcare professions were coded^71^. This includes jobs with job activities entrusted with direct patient care, medical care or healthcare and nursing as well as those tasks in the care and support of people. It also includes all activities in therapy and in the context of disease prevention, detection and treatment. It captures healthcare assurance professions, professions in the sale of medical supplies and medical equipment, professions in nutrition and health counseling, healthcare craftspeople (health and medical technology). Respondents in such jobs were coded 1 and those not as well as those not employed (i.e., unemployed, retired, parental leave, in education) were coded 0. Moreover, we assessed respondents received a diagnosis of a mental illness during their lifetime (such as depression, attention deficit disorder (ADD), attention deficit hyperactivity disorder (ADHD), phobias, dementia, or other mental illnesses) and if they are still in treatment or no longer in treatment. Place of residence (i.e., rural or urban settlement structure) was computed based on postal codes^72^.

### Statistical analysis

We used Pearson’s correlations (which is equivalent to Phi) to examine the strength of associations between the indicators of whether or not individuals self-report knowledge concerning a neurotechnology, whereby *r* >  ± 0.2 indicate small effects, *r* >  ± 0.5 medium effects, and *r* >  ± 0.8 large effects^73^. To examine variation in the binary knowledge indicator (i.e., whether respondents report any knowledge or no knowledge at all), we used Logistic Regression Models and reported odds ratios (*OR*), whereby *OR* above 1 indicate positive effects, *OR* below 1 indicate negative effects, and *OR* equaling 1 indicate no effects. Thereby, *OR* ≥ 1.50 (≤ 0.67) indicate small effects, *OR* ≥ 2 (≤ 0.5) medium effects, and *OR* ≥ 3 (≤ 0.33) large effects^73^. To examine variation in the extent of self-reported knowledge (if at least some knowledge has been reported), we used Ordinary Least Square Regression Models and report standardized coefficients (*β*), whereby, *β* between ± 0.10–0.29 are considered small, *β* of ± 0.30–0.49 medium, and *β* ≥  ± 0.50 large. To indicate the magnitude of the associations between the self-reported knowledge and the independent variables, we report unadjusted odds ratios (*OR*)/*β* for bivariate relations and adjusted *OR*/*β* when controlling for all other independent variables, along with their confidence intervals.

## Results

**Fig. 1:**
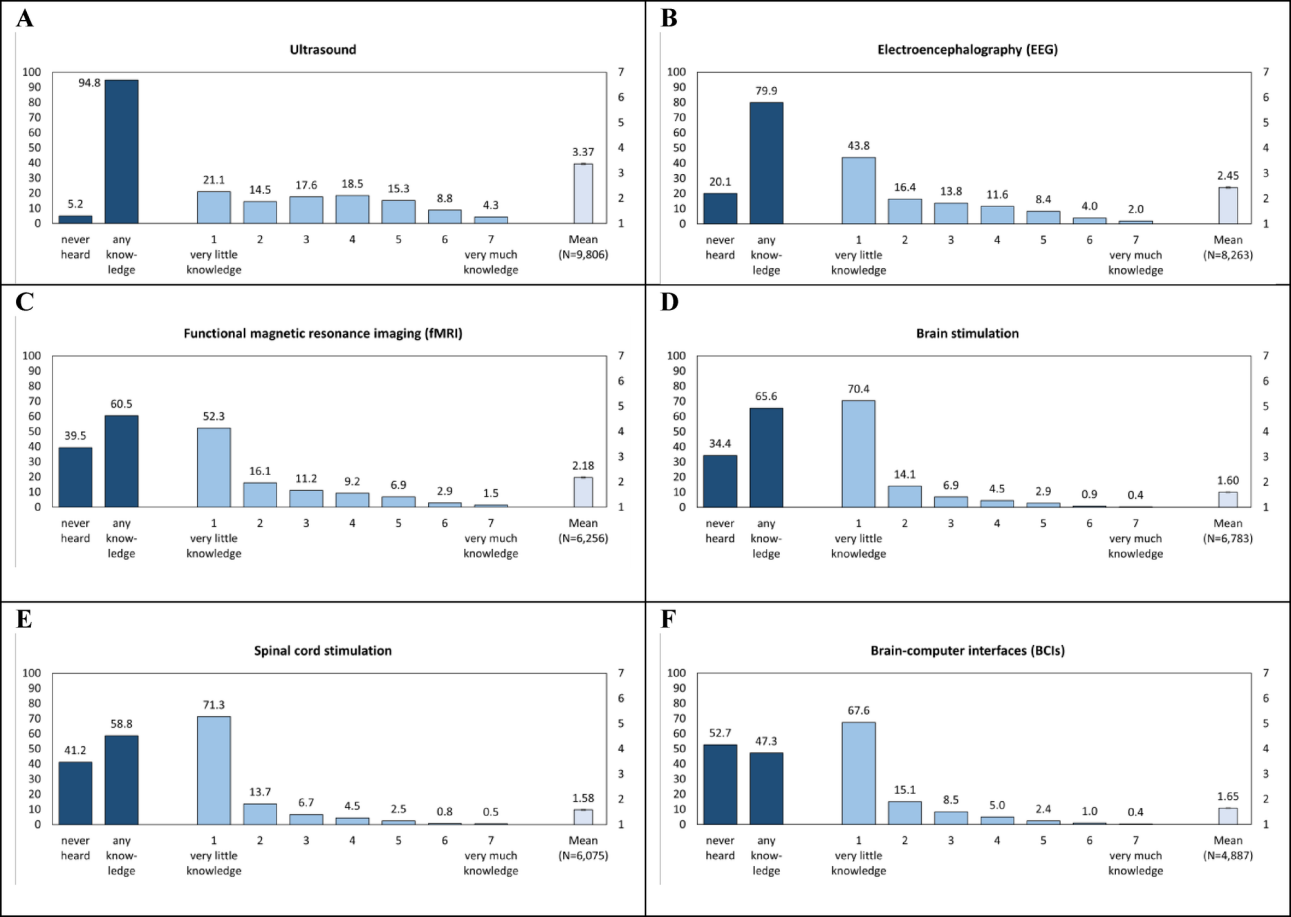
Share of respondents (in %) with and without self-reported knowledge as well as share and mean values (with standard errors) of the extent of self-reported knowledge about neurotechnology (*N* = 10,339)^1^. The number of observations (*N*) refers to “no” and “any knowledge” (dark blue), while the *N* presented for the mean concerning the extent of knowledge (light blue) also refers to bars indicating the extent of knowledge (blue).

Figure 1 shows that almost every respondent (94.8%) has at least some knowledge about ultrasound applications, followed by EEG (79.9%), brain stimulation (65.6%), fMRI (60.5%), spinal cord stimulation (58.8%), while a bit less than half (47.3%) has any knowledge about BCIs. Among those with at least some knowledge, the share of respondents with very little to very high knowledge is much more evenly distributed (although declining towards the higher knowledge) across response options, while for most other neurotechnologies, very little knowledge was the most frequent response—especially regarding brain stimulation (70.4%), spinal cord stimulation (71.3%), and BCIs (67.6%). Moreover, the likelihood of self-reporting any knowledge about different neurotechnologies is positively interrelated—mostly with a small effect size (Table S3). For both stimulation technologies, the strength of interrelation is medium, while the association between ultrasound and BCIs is non-substantial.

**Fig. 2:**
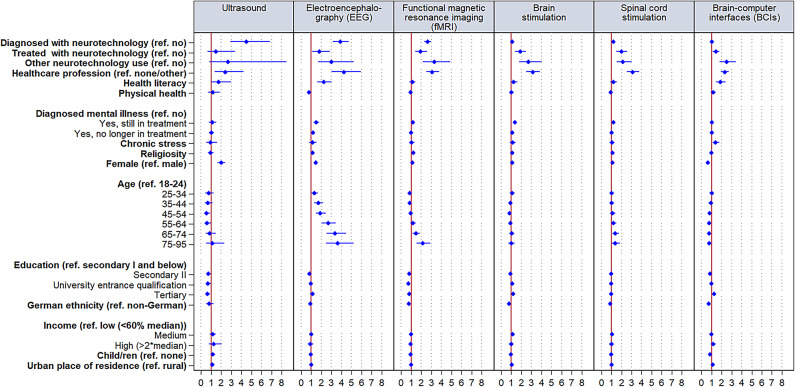
Coefficient plots of self-reporting any knowledge about neurotechnologies (*N* = 10,339). Odds ratios with 95% confidence intervals based on Table S4.* N* = Number of observations.

The multivariate results presented in Fig. 2 and Table S4 show large positive effects of having used neurotechnology for diagnosis on the likelihood of reporting some knowledge concerning ultrasound and EEG, while the effect is of medium size for fMRI, and non-substantial for the other neurotechnologies. Effects for treatment uses are small and positive for EEG, fMRI, brain stimulation, and spinal cord stimulation. Neurotechnology uses for other purposes exert positive medium effects for all neurotechnologies, while the effect is large for fMRI. Moreover, medium effects exist for being in healthcare jobs concerning ultrasound and BCIs, while effects are large for all other neurotechnologies. Higher health literacy is positively associated to the likelihood of reporting some knowledge concerning ultrasound and BCIs, while the effect is medium for EEG.

Effects for indicators of physical and mental health as well as chronic stress and religiosity are non-substantial. Self-identifying as female is positively associated with the likelihood of reporting knowledge of ultrasound and it is negatively associated for BCIs. With the exception of lower likelihoods to self-report knowledge about ultrasound among individuals with university entrance qualification and tertiary education as compared to the lowest educational group, further education effects were non-substantial. As indicated by small effects, the 35–44, 45–54, and 55–64 age groups are less likely to have self-reported knowledge of ultrasound than the youngest group. In addition, the likelihood of reporting some knowledge of EEG seems to generally decrease with age, with up to large effects when comparing the youngest to the two oldest age groups. Regarding fMRI, the oldest age group more likely self-reports knowledge as compared to the youngest age group, as indicated by a medium effect. No substantial effects exist for ethnicity, income, having children, and place of residence.

**Fig. 3:**
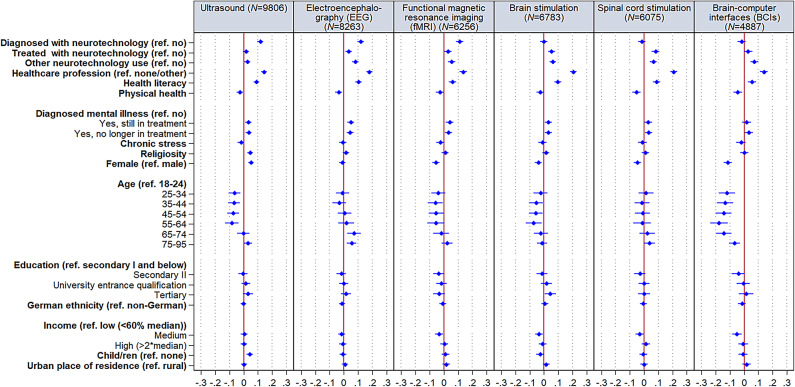
Coefficient plots of self-reported extent of knowledge about neurotechnologies (*N* = 10,339). Standardized regression coefficients with 95% confidence intervals based on Table S5. *N* = Number of observations.

Multivariate results (Fig. 3 and Table S5) show that among individuals reporting some knowledge, having received a diagnosis using ultrasound, EEG, or fMRI increased the extent of the self-reported knowledge with a small effect. Also having a health or care job, was positively associated with more self-reported knowledge concerning all neurotechnologies (small effects), while higher levels of health literacy were only positively associated concerning EEG and brain stimulation knowledge. Being female was negatively associated with the extent of knowledge concerning BCIs. Generally, the oldest age group self-reports less knowledge concerning ultrasound, EEG, and fMRI than several other age groups. Moreover, age group 34–44 self-reports less knowledge concerning EEG than age group 65–74; and the 25 to 74 year olds self-report less knowledge about BCIs than the youngest group. No further substantial variation was observed.

## Discussion

The objectives of this research were, first, to determine how many people in the public lack any knowledge about neurotechnologies; second, to describe the extent of knowledge of those who have heard of such technologies; and third, to examine disparities in knowledge. To this end, we conducted a large nationwide study of the adult population in Germany to identify the public that has never heard of six key neurotechnologies (ultrasound, EEG, fMRI, brain stimulation, spinal cord stimulation, and BCIs) and to describe the extent of self-reported knowledge of those who have heard of it. The results show that the proportion of respondents who say they have never heard of a particular neurotechnology, as well as the extent of knowledge, varies greatly by technology. Most respondents have heard of ultrasound and EEG, probably because these technologies are widely used in diagnostics, with ultrasound in particular being used for a variety of non-neurological applications (e.g., in emergency rooms and obstetrics appointments). Thus, the vast majority of respondents may have first-hand experience with these technologies or have heard of others using them. About 60 to 65 percent have heard of fMRI, brain stimulation, and spinal cord stimulation, and less than 50 percent have heard of BCIs. Among those who have heard of the technologies, most report very little to little knowledge, especially regarding brain stimulation, spinal cord stimulation, and BCIs. Thus, mirroring previous study results on public knowledge that have mostly only investigated a single neurotechnology (especially BCIs)^14,31,36,46^, self-reported knowledge of neurotechnology appears to be generally low.

The results also suggest that the likelihood of self-reporting any knowledge of different neurotechnologies was positively interrelated. This may be indicative, for example, of similar antecedents such as having one or comorbid health conditions for which different neurotechnologies have been used or considered, as well as having professional knowledge about neurotechnology, but also interest in novel technologies. To examine disparities in self-reported knowledge, numerous indicators of, for example, prior experience with neurotechnology, elevated ability to understand such technology, or increased likelihood of current or future need of neurotechnology have been explored. The results suggest that, when controlling for various other factors, prior experience with one or more neurotechnologies is a key factor for whether individuals have heard of the neurotechnology, and our results suggest that mainly experience with diagnosis is somewhat prevalent (18.3% of the respondents), but not with treatment (3.2%), or other use purposes such as enhancement (1.9%). Depending on the neurotechnology, specific prior experiences are relevant, for example, for ultrasound and EEG, a previous diagnosis with a neurotechnology appears very influential, while for fMRI, brain and spinal cord stimulation, and BCIs, other uses are particularly influential. Having been treated with a neurotechnology seems only to substantially increase the likelihoods of having heard of EEG, fMRI, as well as brain and spinal cord stimulation. Among individuals reporting some knowledge, having received a diagnosis using ultrasound, EEG, or fMRI increased the extent of self-reported knowledge. Thus, through prior uses individuals may have been informed (e.g., during medical consultations) or self-educed (e.g., by reading operating instructions of consumer neurotechnology) before, during, or after medical and non-medical applications cf.,^48,74^. This may have increased the likelihood of memorizing the technology, while having little effect on knowledge extent, which may indicate that the information received was limited, too complicated, or got forgotten.

The findings also show that working in a healthcare profession (e.g., direct patient care, medical care, or nursing)—in part strongly—affects the likelihood of knowing each neurotechnology, while also the extent of self-reported knowledge is constantly higher compared to the other respondents. The few studies, using samples of patients and health professionals also found such disparity in having some knowledge^30^. Familiarity with neurotechnology can be seen as a function of professional education^43^ as well as using it themselves and attending or assisting in its use, but also (professional) interest.

In addition to these indicators of experience- and education-based knowledge and familiarity with neurotechnology, health literacy was positively associated to the likelihood of having heard of ultrasound, EEG, and BCIs and to the extent of knowledge concerning EEG and brain stimulation. Individuals with higher health literacy may have had a previous need (e.g., an illness) or interest to inform themselves about medical topics^75–77^. This may have made them more likely to having encountered neurotechnology but also to better comprehend it and related information. Similar to another study on BCIs^14^, respondents with lower formal education also had a low extent of self-reported BCI knowledge, what could be due to lower openness to emerging technology^51^. Higher education was only related to a lower likelihood of having heard of ultrasound, which may warrant further research. Respondents identifying as female more likely reported to having heard of ultrasound, which might be due to gynecological cancer screening examination or pregnancy-related examination. However, they were less likely to report having heard of BCIs, and also report a lower respective extent of knowledge. This latter finding is consistent with a study on the public’s BCI knowledge by Sample et al.^14^. Reporting as female has also been associated with more concerns about BCIs and moral objections against, as well as less openness towards other neurotechnologies^14,30–32,50^—however, we only found this for a single neurotechnology and thus further research is needed.

We found some age-related heterogeneity in knowledge: The youngest age-group was most likely to having heard about ultrasound compared to several other age-groups. There was an age-related increase in the likelihood of having heard of EEG, and such a tendency also existed for fMRI (while only the youngest and oldest age-group substantially differ). Moreover, the extent of BCI knowledge was highest in the youngest age-group. Although moral objections and concerns against different neurotechnologies as well as lower use willingness have been found in prior research^14,32,50^, our findings—with the exception of an age-related lower extent of BCI knowledge—suggest that familiarity with some technologies could be elevated due to age-related diseases^49^. Interestingly, no further disparities existed for other indicators of an elevated likelihood of current or future need of neurotechnology, different values and worldviews, as well as other socio-demographic characteristics. This might be partially due to other variables in the model, for example, a need of technologies may have already resulted in prior use.

### Limitations and directions for future research

One limitation is that self-reported knowledge is a subjective measure, which is prone to self-report biases. Although, such subjective measures are theoretically and practically highly important, future research may also use objective measures to test factual knowledge. While this study provided textual information to describe neurotechnology, future studies may also examine whether pictorial, animated descriptions, or video tutorials affect self-reported knowledge e.g.,^14^. As this research was cross-sectional, longitudinal studies could be beneficial to identify changes in self-reported knowledge over time and causes of such changes as well as consequences thereof (e.g., acceptability or use willingness). Such research may also extend to other socio-cultural contexts to identify possible similarities and differences as well as assess further sources of disparate knowledge (e.g., (social) media consumption or the use of neurotechnology within the social network)^14,40^. Moreover, we used adult samples, but neurotechnology is also used by and on younger individuals, thus making it important to also understand their extent of knowledge as well as disparities between socio-demographic groups. While our study combined a wide variety of neurotechologies to get an overview of the public’s awareness and knowledge, we could not ask additional questions about each family of technologies and its members in a single survey (see also *Methods* section). This may also restrict the comparability between the selected neurotechnologies. However, these data provide a starting point for future studies that may focus on certain neurotechnologies and explore related public knowledge in a more focused and specialized way. Thereby, studies may also differentiate between invasive and noninvasive brain stimulation (e.g., DBS and TMS).

## Conclusion

This study provides insights into the public’s knowledge with both novel and more established neurotechnologies. Results suggest a relatively high familiarity (without deeper knowledge) concerning more established technologies such as ultrasound and EEG, while less than half of the participants have heard of the more novel BCIs. Moreover, they show disparate familiarity concerning several respondent characteristics, such as prior experience for treatment, diagnosis, and other purposes, a background in a healthcare profession, health literacy, and partially gender, age, and education. This study demonstrates associations and potential antecedents of a key variable in health-decision-making models and technology acceptance, as well as of stigmatization. The results suggest a practically relevant, sometimes substantial lack of familiarity and relatively low extents of knowledge about various neurotechnologies, as well as disparities in knowledge across societal groups. While current findings are ambiguous concerning whether knowledge or familiarity reduces concerns and increase acceptance^14,46^, it is important to counteract public misconceptions and false hopes, which are partly fueled by the media^20–22,30^. Therefore, different stakeholders such as clinicians, media representatives, scientists, government agencies, and other professional societies, as well as technology developers should be involved, for example, to develop trainings, provide websites with reliable information, or issue health warnings^16,20,45^.

Increasing public knowledge would also increase patient and consumer autonomy. We encourage future research to further explore the causes of limited familiarity and knowledge gaps, to map misconceptions, and identify ways to improve the public understanding of these increasingly relevant technologies in order to mitigate a potential neurotechnology divide.

## Electronic supplementary material

Below is the link to the electronic supplementary material.

Supplementary Material 1

## Data Availability

The data analyzed are available at ref.^78^.
