# Supplementary material for: Exploring disparities in self-reported knowledge about neurotechnology
Source: Sci Rep. 2025 May 27;15:18488. doi: 10.1038/s41598-025-00460-1 (PMC12116940; doi:10.1038/s41598-025-00460-1)
Supplement: Supplementary file 1 — Supplementary Material 1 [file 41598_2025_460_MOESM1_ESM.pdf]

Sattler, S., Mehlkop, G., Neuhaus, A., Wexler, A., Reiner, P. (2025). Exploring Disparities in Self-Reported Knowledge about Neurotechnology. *Scientific Reports.* https://doi.org/10.1038/s41598-025-00460-1

SUPPLEMENTARY MATERIAL

Pre-Study

Background: Prior to the main study, we conducted a survey experiment to better understand whether providing information about a particular neurotechnology, such as areas of application, how it works, or names of devices in the case of consumer applications (versus just the name of the neurotechnology without further information), affects self-reported knowledge about the neurotechnology. To test with and without information is should inform the main study about whether the substantial investment in survey time (when providing more information) leads to different answers as well as how large this investment could be. This was considered important, given that the names of the technologies are rather complicated and unusual in everyday language, while providing structural and semantic descriptions might cue individuals to help them categorize and memorize what kind of technology is being talked about. Neuropsychological research shows, that subtle perceptual deficits can produce naming problems, even when there is good access to associated semantic knowledge (Humphreys, Price, and Riddoch 1999). Especially, informing about areas of application or function can help remembering whether one has already encountered such technologies (even if not remembering the technology's name). Without information beyond the name of the technology heuristic and biased responses could be more likely, for example, since people who tend to overestimate themselves could be more likely to say that they know a technology without information.

Methods: This web-based experiment has been conducted with an offline-recruited nationwide sample (using a multi-stage random process based on the ADM (Arbeitskreis Deutscher Markt- und Sozialforschungsinstitute e.V.) telephone master sample) with adult respondents in Germany, recruited via the forsa.omninet panel. Therefore, 303 individuals were invited of which 251 (82.8%) provided consent to participate, and 205 (81.7%) completed the study. Our analysis uses answers of 202 respondents due to listwise deletion of missing responses (43.6% women, mean age: 54.60, age range: 18 to 81 years). Completion was incentivized with bonus points (approx. $2.00, convertible to vouchers, a ticket for a charity lottery or a donation to UNICEF). The ethics committee of the University of blinded-for-review approved the study (reference number: blinded-for-review).

Respondents were asked to report on their self-reported knowledge about six neurotechnologies, including ultrasound, EEG, fMRI, brain stimulation, spinal cord stimulation, and BCIs by using the question "How would you rate your knowledge of the following neurotechnologies?". Subsequently they were randomly assigned to either a condition in which they were provided with information about the areas of application, functioning, and names of devices (in case of consumer availability of the technology) (for example, for fMRI: "A strong magnetic field is generated to measure activity in brain regions. This creates a so-called brain scan. The procedure is used, for example, to plan treatment for brain tumors or epilepsy"; see Table S1 for the wording of all six technologies) vs. providing only the name of the neurotechnology.

Table S1: Definitions used during the assessment of self-reported knowledge about neurotechnology

German (original)	English (translated)
**Hirnstimulation** (Dabei werden bestimmte Bereiche des Gehirns durch schwache elektrische Reize oder starke Magnetfelder aktiviert oder gehemmt. Hirnstimulatoren können operativ in den Kopf eingesetzt werden (sogenannte Hirnschrittmacher) oder von außen angewendet werden. Angewendet werden sie z. B. bei Krankheiten wie Migräne, Epilepsie oder Parkinson. Hirnstimulatoren werden aber auch ohne medizinische Notwendigkeit genutzt, z. B. um die Konzentration zu steigern. Bekannte Geräte sind z. B. GoFlow, Focus V3 oder Cefaly.)	**Brain stimulation** (This involves activating or inhibiting certain areas of the brain using weak electrical stimuli or strong magnetic fields. Brain stimulators can be surgically inserted into the head (so-called brain pacemakers) or applied externally. They are used for diseases such as migraine, epilepsy or Parkinson's disease. However, brain stimulators are also used without medical necessity, e.g. to increase concentration. Well-known devices include GoFlow, Focus V3 and Cefaly.)
**Rückenmarkstimulation** (Diese wird z. B. in der Schmerztherapie eingesetzt. Dabei sendet ein implantierter Schmerzschrittmacher geringe elektrische Ströme an das Gehirn und kann so Schmerzen verringern.)	**Spinal cord stimulation** (This is used in pain therapy, for example. An implanted pain pacemaker sends small electrical currents to the brain and can thus reduce pain).
**funktionelle Magnetresonanztomographie (fMRT)** (Dafür wird ein starkes Magnetfeld erzeugt um die Aktivität in Hirnregionen zu messen. So entsteht ein sogenannter Hirnscan. Das Verfahren wird z. B. bei der Therapieplanung bei Hirntumoren oder Epilepsie genutzt.)	**Functional magnetic resonance imaging (fMRI)** (A strong magnetic field is generated to measure activity in brain regions. This creates a so-called brain scan. The procedure is used, for example, to plan treatment for brain tumors or epilepsy).
**Elektroenzephalographie (EEG)** (Dabei wird die elektrische Aktivität des Gehirns auf der Kopfoberfläche gemessen und dann grafisch dargestellt. Anwendungen sind z. B. die Diagnose und Verlaufskontrolle bei Epilepsie, die Überwachung bei Operationen unter Narkose, das Feststellen von Hirntod, oder die Schlafanalyse. Auch verschiedene Formen von Neurofeedback verwenden EEG, hier erhält man Feedback über die Gehirnaktivität. Es gibt auch Anwendungen ohne medizinische Notwendigkeit, z. B. Feedback beim Meditieren oder zur Konzentrationssteigerung. Bekannte Geräte sind z. B. Muse vom Hersteller InteraXon oder Insight und Epoc+ vom Hersteller Emotiv.)	**Electroencephalography (EEG)** (The electrical activity of the brain is measured on the surface of the head and then displayed graphically. Applications include the diagnosis and follow-up of epilepsy, monitoring during operations under anesthesia, determining brain death or sleep analysis. Various forms of neurofeedback also use EEG to provide feedback on brain activity. There are also applications without medical necessity, e.g. feedback during meditation or to increase concentration. Well-known devices include Muse from the manufacturer InteraXon or Insight and Epoc+ from the manufacturer Emotiv).
**Ultraschall** (Dies ist Schall mit Frequenzen außerhalb der Hörfrequenz des Menschen und wird, z. B. bei bildgebenden Verfahren in der Medizin verwendet, wie bei gynäkologischen Untersuchungen oder auch bei Ultraschalltherapie, etwa zur Schmerzbehandlung.)	**Ultrasound** (This is sound with frequencies outside the human hearing frequency and is used, for example, in medical imaging procedures such as gynecological examinations or ultrasound therapy, e.g. for pain treatment).
**Brain-Computer Interfaces (deutsch: Gehirn-Computer-Schnittstellen)** (Dafür werden Elektroden im Kopf (also invasiv) oder Elektroden am Kopf über ein Stirnband befestigt (also nicht-invasiv). Brain-Computer Interfaces erlauben die Steuerung von Geräten durch eine Verbindung zwischen dem Gehirn und einem Computer, z. B. können Prothesen mit Hilfe von Gedanken gesteuert werden oder auch Computerspiele. Bekannte nicht-medizinische Hersteller sind z. B. Neuralink, Neurable, Emotiv, NextMind, Kernel oder Dreem.)	**Brain-computer interfaces (German: Gehirn-Computer-Schnittstellen)** (electrodes are attached to the head (i.e. invasive) or electrodes are attached to the head via a headband (i.e. non-invasive). Brain-computer interfaces allow devices to be controlled via a connection between the brain and a computer, e.g. prostheses can be controlled with the help of thoughts or computer games. Well-known non-medical manufacturers include Neuralink, Neurable, Emotiv, NextMind, Kernel and Dreem).

Respondents indicated their knowledge using a scale from “never heard of it” [value 0], “very little knowledge” [1] to “very much knowledge” [7]. Participants who chose not to respond to this question could choose a non-scaled option (“no response”). For each neurotechnology, two

indicators have been created to first, examine factors influencing the complete absence of knowledge [0] vs. any knowledge [1] and to second, examine factors influencing the extent of knowledge indicated by values ranging from 1 to 7, excluding respondents indicating no knowledge at all.

Results: Pearson's correlations (which is equivalent to Phi) in Table S2 show that the effect of the experimental treatment (i.e., providing no information vs. providing information) on whether or not individuals self-report knowledge were non-substantial – all coefficients were below the benchmark of |0.2| which would indicate small effects (Sullivan and Feinn 2012). The *Cohen's d* values show that providing knowledge had non-substantial effects concerning the extent of self-reported knowledge – all values were below the benchmark of |0.2| which would indicate small effects (Sullivan and Feinn 2012). The only exception exists for BCIs, where a small effect size indicates a higher self-reported knowledge in the information condition. Moreover, the mean response time in the condition with information almost doubled to 62.23 seconds (*95%-CI*=53.99;70.47; *N*=103) compared to the condition without information (34.97 seconds, *95%-CI*=31.67;38.27; *N*=99). This difference is of large size (*Cohen's d*=0.84; *95%-CI*=0.55;1.13).

Table S2: Effect sizes for experimental treatment effects

	Pearson's correlation (for no vs. any knowledge, *N* in brackets)	***Cohen's d*** (for the extent of knowledge, *N* in brackets)
**1. Ultrasound**	-0.030 [N_i=103; N_n=99]	0.083 [N_i=98; N_n=95]
**2. Electroencephalography (EEG)**	-0.001 [N_i=103; N_n=99]	0.003 [N_i=82; N_n=81]
**3. Functional magnetic resonance imaging (fMRI)**	-0.033 [N_i=103; N_n=99]	-0.070 [N_i=70; N_n=60]
**4. Brain stimulation**	-0.049 [N_i=103; N_n=99]	0.108 [N_i=68; N_n=63]
**5. Spinal cord stimulation**	-0.024 [N_i=103; N_n=99]	0.067 [N_i=63; N_n=60]
**6. Brain-computer interfaces (BCIs)**	-0.034 [N_i=103; N_n=99]	*0.267* [N_i=52; N_n=56]

Notes: Small effects (d>±0.2) are displayed in *italics*. The subscript *i* for the number of observations (*N*) indicates the information treatment and the subscript *n* the no information treatment.

Conclusion: This pre-study investigated whether providing structural and semantic descriptions of neurotechnologies – with names that are rather complicated and unusual in everyday language – affects self-reported knowledge. The limited impact of providing such information on whether respondents remembered if they had heard about the six neurotechnologies, as well as on the extent of knowledge suggests that investing survey time in explaining neurotechnology in this form may not always be advisable. The results may also indicate that respondents can categorize themselves without such cues.

Table S3: Pairwise Pearson's correlations of the self-reported knowledge with and without self-reported knowledge about neurotechnology (*N*=10,339)

	**1.**	**2.**	**3.**	**4.**	**5.**
**1. Ultrasound**					
**2. Electroencephalography (EEG)**	*0.387*				
**3. Functional magnetic resonance imaging (fMRI)**	*0.257*	*0.458*			
**4. Brain stimulation**	*0.231*	*0.317*	*0.388*		
**5. Spinal cord stimulation**	*0.222*	*0.330*	*0.450*	***0.685***	
**6. Brain-computer interfaces (BCIs)**	0.147	*0.247*	*0.337*	*0.456*	*0.423*

Notes: Small effects (*r*>±0.2) are displayed in *italics* and medium effects (*r*>±0.5) in ***bolded italics***.

Table S4: Multivariate logistic regression of self-reported knowledge about neurotechnologies (*N*=10,339)

	**Ultrasound**	**Electro-encephalography (EEG)**	**Functional magnetic resonance imaging (fMRI)**	**Brain stimulation**	**Spinal cord stimulation**	**Brain-computer interfaces (BCIs)**
**Treated with neurotechnology** (ref. no)	1.47 [0.64;3.39]	*1.79* [1.13;2.85]	*1.90* [1.40;2.58]	*1.85* [1.40;2.46]	*1.97* [1.51;2.57]	1.40 [1.10;1.77]
**Diagnosed with neurotechnology** (ref. no)	**4.46** [2.91;6.85]	**3.87** [3.17;4.72]	***2.61*** [2.31;2.95]	1.07 [0.96;1.20]	1.17 [1.05;1.30]	1.03 [0.93;1.15]
**Other neurotechnology use** (ref. no)	***2.68*** [0.84;8.49]	***2.97*** [1.70;5.20]	**3.26** [2.18;4.88]	***2.67*** [1.78;4.02]	***2.13*** [1.51;3.00]	***2.48*** [1.79;3.44]
**Healthcare profession** (ref. none/other)	***2.41*** [1.37;4.24]	**4.24** [3.03;5.94]	**3.07** [2.51;3.74]	**3.09** [2.49;3.84]	**3.09** [2.54;3.75]	***2.30*** [1.95;2.72]
**Health literacy**	*1.74* [1.01;2.99]	***2.21*** [1.62;3.02]	1.09 [0.84;1.41]	1.22 [0.94;1.59]	1.19 [0.93;1.53]	*1.87* [1.45;2.41]
**Physical health**	1.15 [0.72;1.85]	0.78 [0.60;1.03]	0.93 [0.75;1.16]	0.99 [0.79;1.23]	0.90 [0.72;1.11]	1.16 [0.94;1.43]
**Diagnosed mental illness** (ref. no)						
- Yes, still in treatment	1.09 [0.80;1.49]	1.48 [1.23;1.78]	1.15 [1.00;1.32]	1.31 [1.14;1.51]	1.17 [1.03;1.34]	1.02 [0.89;1.16]
- Yes, no longer in treatment	1.01 [0.81;1.26]	1.17 [1.03;1.33]	0.97 [0.88;1.07]	1.06 [0.96;1.18]	0.99 [0.90;1.10]	1.03 [0.93;1.14]
**Chronic stress**	0.92 [0.54;1.58]	1.11 [0.82;1.51]	1.03 [0.80;1.31]	1.11 [0.87;1.43]	1.03 [0.81;1.32]	1.38 [1.08;1.75]
**Religiosity**	0.92 [0.69;1.23]	1.14 [0.97;1.35]	1.19 [1.04;1.36]	1.07 [0.93;1.22]	1.10 [0.97;1.25]	0.95 [0.84;1.08]
**Female** (ref. male)	1.98 [1.63;2.40]	1.44 [1.29;1.59]	1.12 [1.03;1.22]	1.08 [0.99;1.18]	1.10 [1.01;1.19]	*0.62* [0.57;0.68]
**Age** (ref. 18-24)						
- 25-34	0.74 [0.44;1.26]	1.29 [1.02;1.65]	0.82 [0.66;1.03]	1.06 [0.84;1.33]	1.01 [0.82;1.26]	1.00 [0.80;1.24]
- 35-44	*0.66* [0.38;1.13]	*1.70* [1.32;2.20]	0.84 [0.66;1.05]	0.88 [0.69;1.12]	1.01 [0.80;1.26]	0.91 [0.72;1.13]
- 45-54	*0.52* [0.30;0.90]	*1.89* [1.46;2.46]	0.93 [0.73;1.17]	0.78 [0.62;0.99]	1.10 [0.87;1.38]	0.80 [0.64;1.01]
- 55-64	*0.56* [0.33;0.95]	***2.66*** [2.06;3.44]	1.15 [0.92;1.45]	0.88 [0.70;1.11]	1.20 [0.96;1.50]	0.77 [0.61;0.96]
- 65-74	0.82 [0.46;1.46]	**3.36** [2.53;4.47]	1.45 [1.13;1.86]	1.02 [0.79;1.30]	1.37 [1.08;1.73]	0.75 [0.59;0.95]
- 75-95	1.10 [0.52;2.30]	**3.61** [2.51;5.20]	***2.10*** [1.54;2.87]	0.99 [0.73;1.33]	1.38 [1.03;1.84]	0.76 [0.57;1.01]
**Education** (ref. secondary I and below)						
- Secondary II	0.72 [0.51;1.02]	0.83 [0.69;1.00]	0.77 [0.66;0.90]	0.90 [0.77;1.04]	0.94 [0.81;1.08]	0.84 [0.73;0.97]
- University entrance qualification	*0.67* [0.47;0.98]	0.93 [0.76;1.13]	0.70 [0.60;0.83]	1.07 [0.91;1.25]	0.96 [0.82;1.12]	0.97 [0.83;1.13]
- Tertiary	*0.63* [0.45;0.90]	1.11 [0.92;1.34]	0.77 [0.66;0.90]	1.14 [0.98;1.32]	0.96 [0.83;1.11]	1.26 [1.09;1.45]
**German ethnicity** (ref. non-German)	0.80 [0.51;1.24]	0.90 [0.72;1.14]	0.75 [0.62;0.91]	0.75 [0.62;0.91]	0.87 [0.73;1.04]	0.69 [0.58;0.82]
**Income** (ref. low (<60% median))						
- Medium	1.14 [0.88;1.47]	1.01 [0.87;1.17]	0.97 [0.86;1.10]	1.12 [0.99;1.26]	1.04 [0.93;1.17]	0.96 [0.86;1.08]
- High (>2*median)	1.25 [0.78;2.01]	0.91 [0.70;1.19]	0.92 [0.73;1.14]	0.96 [0.77;1.21]	0.98 [0.79;1.22]	1.13 [0.91;1.40]
**Child/ren** (ref. none)	1.17 [0.96;1.42]	0.96 [0.85;1.07]	0.94 [0.85;1.03]	0.96 [0.87;1.05]	0.96 [0.88;1.05]	0.85 [0.77;0.93]
**Urban place of residence** (ref. rural)	1.10 [0.90;1.33]	0.98 [0.88;1.10]	0.97 [0.88;1.06]	1.04 [0.95;1.14]	1.01 [0.92;1.10]	1.12 [1.02;1.22]

Notes: *N*=Number of observations. Odds Ratios with 95% confidence intervals in parentheses. Small effects (*OR*≥1.50 and ≤0.67) are displayed in *italics*, medium effects (*OR*≥2 and ≤0.5) in ***bolded italics***, and large effects (*OR*≥3 and ≤0.33) in **bold**.

Table S5: Multivariate linear regression of self-reported knowledge about neurotechnologies

	**Ultrasound (*N*=9,806)**	**Electro-encephalography (EEG) (*N*=8,263)**	**Functional magnetic resonance imaging (fMRI) (*N*=6,256)**	**Brain stimulation (*N*=6,783)**	**Spinal cord stimulation (*N*=6,075)**	**Brain-computer interfaces (BCIs) (*N*=4,887)**
**Treated with neurotechnology** (ref. no)	0.02 [-0.04;0.36]	0.03 [0.11;0.48]	0.03 [0.03;0.41]	0.05 [0.17;0.45]	0.08 [0.31;0.59]	0.03 [-0.01;0.33]
**Diagnosed with neurotechnology** (ref. no)	*0.12* [0.43;0.61]	*0.12* [0.39;0.57]	*0.11* [0.31;0.49]	-0.00 [-0.07;0.06]	-0.01 [-0.11;0.03]	-0.02 [-0.13;0.04]
**Other neurotechnology use** (ref. no)	0.03 [0.07;0.57]	0.08 [0.67;1.14]	0.05 [0.29;0.77]	0.06 [0.28;0.62]	0.06 [0.29;0.64]	0.07 [0.30;0.68]
**Healthcare profession** (ref. none/other)	*0.14* [0.87;1.14]	*0.18* [0.97;1.23]	*0.14* [0.61;0.88]	*0.21* [0.74;0.93]	*0.21* [0.71;0.91]	*0.14* [0.44;0.67]
**Health literacy**	0.09 [0.75;1.18]	*0.10* [0.83;1.26]	0.06 [0.32;0.80]	*0.10* [0.51;0.85]	0.09 [0.43;0.78]	0.05 [0.19;0.59]
**Physical health**	-0.03 [-0.41;-0.05]	-0.04 [-0.45;-0.09]	-0.03 [-0.39;0.01]	-0.02 [-0.27;0.01]	-0.05 [-0.42;-0.13]	-0.04 [-0.40;-0.07]
**Diagnosed mental illness** (ref. no)						
- Yes, still in treatment	0.03 [0.06;0.29]	0.05 [0.12;0.34]	0.04 [0.06;0.30]	0.03 [0.02;0.19]	0.03 [0.01;0.19]	0.02 [-0.05;0.16]
- Yes, no longer in treatment	0.04 [0.06;0.23]	0.04 [0.09;0.25]	0.03 [0.02;0.21]	0.03 [0.01;0.14]	0.03 [0.01;0.15]	0.03 [0.01;0.17]
**Chronic stress**	-0.02 [-0.37;0.04]	-0.01 [-0.27;0.15]	-0.02 [-0.42;0.04]	-0.01 [-0.23;0.09]	-0.01 [-0.23;0.10]	-0.02 [-0.32;0.07]
**Religiosity**	0.05 [0.15;0.37]	0.02 [-0.03;0.19]	0.01 [-0.08;0.17]	0.01 [-0.03;0.14]	0.01 [-0.05;0.13]	0.00 [-0.10;0.11]
**Female** (ref. male)	0.05 [0.11;0.25]	-0.01 [-0.11;0.04]	-0.05 [-0.25;-0.09]	-0.04 [-0.14;-0.03]	-0.05 [-0.16;-0.05]	*-0.11* [-0.33;-0.20]
**Age** (ref. 18-24)						
- 25-34	-0.06 [-0.49;-0.11]	-0.01 [-0.25;0.16]	-0.04 [-0.37;0.05]	-0.02 [-0.21;0.07]	0.01 [-0.11;0.19]	*-0.12* [-0.50;-0.18]
- 35-44	-0.07 [-0.51;-0.12]	-0.03 [-0.35;0.07]	-0.06 [-0.48;-0.04]	-0.06 [-0.32;-0.02]	-0.01 [-0.20;0.12]	*-0.13* [-0.57;-0.23]
- 45-54	-0.08 [-0.56;-0.17]	0.01 [-0.18;0.24]	-0.05 [-0.46;-0.02]	-0.06 [-0.33;-0.03]	-0.01 [-0.19;0.13]	*-0.14* [-0.63;-0.28]
- 55-64	-0.08 [-0.54;-0.15]	0.02 [-0.14;0.27]	-0.05 [-0.41;0.02]	-0.07 [-0.34;-0.04]	-0.01 [-0.19;0.12]	*-0.18* [-0.65;-0.31]
- 65-74	-0.00 [-0.22;0.19]	0.07 [0.10;0.54]	-0.02 [-0.31;0.15]	-0.02 [-0.23;0.08]	0.02 [-0.10;0.23]	*-0.14* [-0.67;-0.30]
- 75-95	0.03 [-0.00;0.49]	0.06 [0.17;0.68]	0.02 [-0.11;0.42]	-0.01 [-0.27;0.11]	0.04 [-0.00;0.40]	-0.07 [-0.62;-0.17]
**Education** (ref. secondary I and below)						
- Secondary II	-0.01 [-0.14;0.10]	-0.02 [-0.18;0.06]	-0.04 [-0.25;0.00]	-0.01 [-0.13;0.06]	-0.03 [-0.17;0.03]	-0.04 [-0.23;0.01]
- University entrance qualification	0.01 [-0.07;0.20]	-0.00 [-0.14;0.13]	-0.02 [-0.21;0.08]	0.02 [-0.05;0.15]	0.00 [-0.10;0.11]	-0.01 [-0.14;0.11]
- Tertiary	0.03 [-0.01;0.23]	0.01 [-0.07;0.17]	-0.03 [-0.24;0.02]	0.04 [0.01;0.20]	-0.00 [-0.10;0.10]	0.01 [-0.08;0.15]
**German ethnicity** (ref. non-German)	-0.00 [-0.16;0.14]	-0.01 [-0.24;0.06]	-0.01 [-0.21;0.11]	0.01 [-0.08;0.14]	-0.01 [-0.14;0.09]	-0.02 [-0.20;0.06]
**Income** (ref. low (<60% median))						
- Medium	0.00 [-0.09;0.11]	-0.02 [-0.16;0.04]	-0.03 [-0.24;-0.02]	-0.03 [-0.18;-0.02]	-0.03 [-0.17;-0.01]	-0.05 [-0.24;-0.05]
- High (>2*median)	0.00 [-0.17;0.21]	-0.01 [-0.24;0.14]	0.00 [-0.19;0.23]	-0.01 [-0.20;0.09]	0.01 [-0.09;0.22]	-0.01 [-0.20;0.13]
**Child/ren** (ref. none)	0.04 [0.07;0.23]	-0.01 [-0.10;0.06]	0.01 [-0.05;0.12]	-0.03 [-0.12;-0.00]	-0.01 [-0.08;0.05]	-0.01 [-0.09;0.05]
**Urban place of residence** (ref. rural)	0.00 [-0.07;0.09]	0.01 [-0.05;0.10]	0.02 [-0.03;0.14]	0.02 [-0.02;0.10]	0.00 [-0.06;0.06]	0.02 [-0.03;0.11]

Notes: *N*=Number of observations; Standardized coefficients with 95% confidence intervals in parentheses. Small effects (β±0.10–0.29) are displayed in *italics*.
